# Family History and Breast Cancer Hormone Receptor Status in a Spanish Cohort

**DOI:** 10.1371/journal.pone.0029459

**Published:** 2012-01-06

**Authors:** Xuejuan Jiang, Jose Esteban Castelao, Elisabet Chavez-Uribe, Beatriz Fernandez Rodriguez, Catuxa Celeiro Muñoz, Carmen M. Redondo, Maite Peña Fernandez, Alejandro Novo Dominguez, Carina Doris Pereira, María Elena Martínez, Tomás García-Caballero, Máximo Fraga Rodriguez, José Antúnez, Angel Carracedo, Jerónimo Forteza-Vila, Manuela Gago-Dominguez

**Affiliations:** 1 Department of Preventive Medicine, University of Southern California, Los Angeles, California, United States of America; 2 Oncology and Genetics Unit, Complejo Hospitalario Universitario de Vigo, Vigo Spain; 3 Complejo Hospitalario Universitario de Santiago de Compostela, Santiago De Compostela, Spain; 4 Arizona Cancer Center, University of Arizona, Tucson, Arizona, United States of America; 5 Galician Foundation of Genomic Medicine, Santiago de Compostela, Spain; Health Canada, Canada

## Abstract

**Background:**

Breast cancer is a heterogenous disease that impacts racial/ethnic groups differently. Differences in genetic composition, lifestyles, reproductive factors, or environmental exposures may contribute to the differential presentation of breast cancer among Hispanic women.

**Materials and Methods:**

A population-based study was conducted in the city of Santiago de Compostela, Spain. A total of 645 women diagnosed with operable invasive breast cancer between 1992 and 2005 participated in the study. Data on demographics, breast cancer risk factors, and clinico-pathological characteristics of the tumors were collected. Hormone receptor negative tumors were compared with hormone receptor postive tumors on their clinico-pathological characteristics as well as risk factor profiles.

**Results:**

Among the 645 breast cancer patients, 78% were estrogen receptor-positive (ER+) or progesterone receptor-positive (PR+), and 22% were ER−&PR−. Women with a family history of breast cancer were more likely to have ER−&PR− tumors than women without a family history (Odds ratio, 1.43; 95% confidence interval, 0.91–2.26). This association was limited to cancers diagnosed before age 50 (Odds ratio, 2.79; 95% confidence interval, 1.34–5.81).

**Conclusions:**

An increased proportion of ER−&PR− breast cancer was observed among younger Spanish women with a family history of the disease.

## Introduction

Breast cancer is a heterogenous disease with a range of morphological phenotypes and histopathological subtypes with distinct prognostic characteristics. It has been shown that women diagnosed with estrogen receptor-positive (ER+)/progesterone receptor-positive (PR+) tumors are more responsive to hormonal treatment and have a better prognosis than those diagnosed with estrogen receptor-negative (ER−)/progesterone receptor negative (PR−) tumors, indicating etiologic heterogeneity of hormone-receptor defined subtypes of breast cancer [1]. Consistently, disparate risk factor profiles for breast cancer according to ER and PR status have been reported [2]; however, risks associated with family history of breast cancer do not seem to differ by receptor status. In a recent study, Hines et al. [3] reported that family history (FH) was significantly associated with an increased risk of both ER+ and ER− breast cancers among non-Hispanic White (NHW) women; however, among Hispanic women, having a family history was associated with an increased risk of ER− but not ER+e tumors, indicating a distinct pattern of breast cancer among Hispanics.

Breast cancer impacts differently among each racial/ethnic group in the United States [4], [5], [6], [7]. Compared with NHW women, Hispanic women have a lower incidence rate of breast cancer; however, once diagnosed with this disease they are more likely of dying from it. Such difference in survival may be attributed to socioeconomic factors and/or differences in access to screening and treatment [8]. However, studies [9], [10] have found that despite equal access to health care services, differences persist in the presentation of Hispanic women with breast cancer compared with NHW women, indicating a biologic basis for the racial/ethnic differences. These differences may result from racial/ethnic differences in genetic composition, lifestyles, reproductive factors, or environmental exposures [10].

Here we describe the characteristics of breast cancer subtypes defined by ER and PR status and assess the associations between FH and ER and PR status in a series of female breast cancer patients in Spain. To our knowledge, this study represents one of the first studies to explore these relationships in a large population of Spanish women.

## Materials and Methods

### Ethics

We obtained ethics approval for our study from the Comité Ético de Investigación de Galicia associated with the Complexo Hospitalario Universitario de Santiago from where all participants were recruited. This study was conducted according to the Spanish law including adherence to the Helsinki Principles of 1975, as revised in 1983. Verbal informed consent, which was used in majority of research studies at the time our study was initiated, was specifically approved by the Comité Ético de Investigación de Galicia. The information sheet was dated to document each subject's consent.

### Study Population

As a part of the Breast Oncology Galician Network (BREOGAN), a population-based study was conducted in the city of Santiago de Compostela, Spain within a geographically defined health region that covers aproximately 500,000 inhabitants. The study involved 663 women with operable invasive breast cancer diagnosed and treated between April 1991 and December 2005 at the Clinical University Hospital of Santiago de Compostela (Santiago de Compostela, Spain) [11].

### Data Collection

Risk factor and clinical information were collected in two ways. Data on demographics, FH, reproductive history and other variables were collected through a risk factor questionnaire. Clinical and histopathological data were abstracted from medical records by trained physicians. FH was defined as self-reported history of breast cancer in any first- or second-degree relatives. Information on FH was available for 645 of the 663 breast cancer patients with known joint ER and PR status.

### Clinico-Pathological Data

Immunohistochemistry (IHC) analyses on paraffin-embedded material were performed to determine the status of ER, PR, MIB-1, and P53. In every tumor, 4-µm histological sections were cut and stained with hematoxylin and eosin for histopathological examination according to the criteria of the World Health Organization [12]. Histological grading was evaluated using the Nottingham modification of the Bloom-Richardson system [13]. IHC analysis on paraffin-embedded material was performed using antibodies for ER (clone 6F11, dilution 1∶50, water bath; Novocastra, Newcastle-upon- Tyne, UK), PR (clone PgR 636, dilution 1∶50, water bath; Dako, Glostrup, Denmark), MIB-1 (clone Ki-67, dilution 1∶200, water bath; Dako), and p53 (clone DO7, dilution 1∶20, water bath; Novocastra). A peroxidase-conjugated labeled dextran polymer was used as detection system (EnVision®, Peroxidase/DAB; Dako). Negative and positive controls were concurrently run for all antibodies with satisfactory results. Cells were considered immunopositive when diffuse or dot-like nuclear staining was observed regardless of the intensity of the staining; only nuclear immunoreactivity was considered specific. The number of positive cells was counted by two different observers independently. Whenever necessary, a consensus was reached using a double-headed microscope. ER, PR and p53 were considered positive when the percent of immunostained nuclei was ≥10%. MIB-1 results were classified as low (≤17%), moderate (18–34%) or high (≥35%).

#### Flow Cytometry and Karyometry

Flow cytometry analysis was performed on fresh material from specimens obtained at the time of surgery as previously reported [14]. The primary medical use of flow cytometry is the indirect measurement of intracellular DNA content. Measurement of the amount of DNA content in tumor cells gives an indication of cell proliferation, as well as cells with an abnormal amount of DNA, and thus may be of prognostic value in cancer studies. SPF is defined as the percentage of cells in phase S, in which the cell duplicates its DNA. DNA index (DI) is defined as the ratio of the G0/G1 channel number of tumor cells to the G0/G1 channel number of diploid cells. Tumor samples were classified into two categories in relation to DI: diploid and near diploid (DI = 0.96−1.15), aneuploid (DI>1.15 or DI<0.96).

Karyometry was carried out at a magnification of ×400 using a light microscope with an eyepiece equipped with a micrometer grid. Details of procedures have been described in a previous publication [11].

### Statistical Analyses

Breast cancer was classified into two categories based on ER and PR status: ER-positive or PR-positive (ER+/PR+) tumors versus ER-negative and PR-negative (ER−&PR−) tumors. Case-only analysis was conducted. Characteristics between breast cancer subtypes were compared using univariate methods, i.e. t-tests for continuous variables and χ^2^ tests for categorical variables (fisher's exact tests were used where sample sizes were small). Similar statistical methods were used to compare cases with and without a family history. Multivariate logistic regression was used to estimate odds ratios (ORs), 95% confidence intervals (CIs), and *P* values for associations between family history and breast cancer subtypes while simultaneously controlling for age. Outcome (dependent) variables were breast cancer subtypes defined by ER and PR status as well as DI, and explanatory variable was family history of breast cancer among any first- or second-degree relatives (present vs. absent).

All statistical analyses were performed using the SAS 9.2 statistical software (SAS Institute Inc., Cary, NC). All reported test significance levels (P values) were two-sided.

## Results

A total of 645 breast cancer patients with known joint ER and PR status were identified. Among them, 22% were ER−&PR− and 78% were ER+/PR+ (Table 1). The age of these patients ranged from 25 to 85 years, with a mean of 59 years. Compared to women with ER+/PR+ tumors, women with ER−&PR− tumors were similar in age at diagnosis and tumor size, but were more likely to have medullary carcinoma. ER−&PR− tumors were also more likely to be high grade, highly proliferative (based on S-phase fraction and MIB-1 level), P53 positive, and aneuploid with larger nuclear areas and perimeters; however, data on grade, MIB-1 and P53 expression were missing for at least 106 patients.

**Table 1:** Clinicopathological, Karyometric, and Immunohistochemical Characteristics of Breast Cancer Cases and by ER and PR status.

	All	ER+/PR+	ER−&PR−	
	(N = 645)	(N = 504)	(N = 141)	*P_ER−&PR− vs. ER+/PR+_* 1
**Age at diagnosis (years)**, ±SD^4^	59±14	59±14	59±16	*0.67*
Median±interquartile range	60±21	60±21	57±24	*0.66*
<50, n (%)	467 (72%)	366 (73%)	101 (72%)	*0.83*
≥50, n (%)	178 (28%)	138 (27%)	40 (28%)	
**Tumor size (cm)**, ±SD^4^	3.5±3.2	3.4±3.3	3.6±2.5	*0.48*
**Histological type** 2, n (%)				*<0.001*
Invasive ductal carcinoma	531 (83%)	420 (84%)	111 (79%)	
Invasive lobular carcinoma	57 (9%)	49 (10%)	8 (6%)	
Invasive medullary carcinoma	21 (3%)	5 (1%)	16 (11%)	
Other	34 (5%)	29 (6%)	5 (4%)	
**Axillary lymph node metastases** 2, n (%)	338 (53%)	274 (55%)	64 (45%)	*0.041*
**Microscopic grade** 2, n (%)				*<0.001*
Grade I	117 (22%)	110 (25%)	7 (7%)	
Grade II	305 (57%)	263 (61%)	42 (39%)	
Grade III	117 (22%)	59 (14%)	58 (54%)	
**S-phase fraction**, n (%)				*<0.001*
Low (≤5%)	252 (39%)	224 (44%)	28 (20%)	
Moderate (>5–10%)	248 (38%)	190 (38%)	58 (41%)	
High (>10%)	145 (22%)	90 (18%)	55 (39%)	
**MIB-1** 2, n (%)				*<0.001*
Low (≤17%)	155 (30%)	148 (37%)	7 (6%)	
Moderate (18–34%)	201 (38%)	167 (42%)	34 (28%)	
High (≥35%)	168 (32%)	86 (21%)	82 (67%)	
**P53 expression** 2, n (%)				*0.009*
Negative	257 (62%)	207 (66%)	50 (51%)	
Positive	158 (38%)	109 (34%)	49 (49%)	
**Ploidy** 2, n (%)				*0.049*
Diploid & near-diploid	199 (31%)	167 (33%)	32 (23%)	
Hyperploid	411 (64%)	309 (61%)	102 (72%)	
Hypoploid	34 (5%)	27 (5%)	7 (5%)	
**Nuclear area** 3 (µm^2^), ±SD^4^	104±46	99±42	121±56	*0.001*
**Perimeter** 3(µm), ±SD^4^	70±14	68±13	75±17	*0.002*
**Spherical** 3 (%), ±SD^4^	59±15	60±14	55±17	*0.014*
**Oval** 3 (%), ±SD^4^	31±7	31±7	31±8	*0.96*
**Cylindrical** 3 (%), ±SD^4^	11±18	10±19	14±14	*0.035*

1 Ps for categorical variables were estimated from χ^2^ tests. P for the comparison of median ages was esimated from Wilcoxon Rank-Sum test and Ps for other continuous variables were estimated from t-tests.

2 Histology, lymph node metastasis, grade, MIB1, P53 expression and DNA ploidy index, were unknown for 2, 7, 106, 121, 230 and 1 cases, respectively.

3 Data on nuclear are, perimeter and DNA shape were available for only 353 cases.

4 Mean ±standard deviation.

We also compared tumor characteristis between breast cancer patients with a positve family history and those without (Table 2). Women with a family history were more likely to be diagnosed at an earlier age and were more likely to be pre- or peri-menopausal, but were less likely to have large tumors.

**Table 2:** Clinicopathological, karyometric, and immunohistochemical characteristics of breast cancer by family history.

	No family history	Family history	
	(n = 520)	(n = 125)	*P* 1
**Age at diagnosis (years)**, ±SD^4^	60±14	54±14	*<0.001*
Median±interquartile range	61±21	50±23	*<0.001*
<50, n (%)	117 (22%)	61 (49%)	*<0.001*
≥50, n (%)	403 (78%)	64 (51%)	
**Tumor size (cm)**, ±SD^4^	3.6±3.4	3.0±1.6	*0.007*
**Histological type** 2, n (%)			*0.082*
Invasive ductal carcinoma	421 (81%)	110 (89%)	
Invasive lobular carcinoma	50 (10%)	7 (6%)	
Invasive medullary carcinoma	16 (3%)	5 (4%)	
Other	32 (6%)	2 (2%)	
**Axillary lymph node metastases** 2, n (%)	270 (53%)	68 (54%)	*0.76*
**Microscopic grade** 2, n (%)			*<0.13*
Grade I	99 (23%)	18 (16%)	
Grade II	232 (54%)	73(65%)	
Grade III	95 (22%)	22 (19%)	
**S-phase fraction**, n (%)			*0.30*
Low (≤5%)	210 (40%)	42 (34%)	
Moderate (>5–10%)	193 (37%)	55 (44%)	
High (>10%)	117 (23%)	28 (22%)	
**MIB-1** 2, n (%)			*0.22*
Low (≤17%)	128 (31%)	27 (26%)	
Moderate (18–34%)	164 (39%)	37 (35%)	
High (≥35%)	127 (30%)	41 (39%)	
**Ploidy** 2, n (%)			*0.78*
Diploid & near-diploid	160 (31%)	39 (31%)	
Hyperploid	330 (64%)	81 (65%)	
Hypoploid	29 (6%)	5 (4%)	
**P53 expression** 2, n (%)			*0.089*
Negative	203 (60%)	54 (71%)	
Positive	136 (40%)	22 (29%)	
**Nuclear area** 3 (µm^2^), ±SD^4^	105±46	96±44	*0.18*
**Perimeter** 3(µm), ±SD^4^	70±15	67±13	*0.11*
**Spherical** 3 (%), ±SD^4^	58±15	63±14	*0.032*
**Oval** 3 (%), ±SD^4^	31±7	30±7	*0.37*
**Cylindrical** 3 (%), ±SD^4^	12±19	7±9	*0.005*

1 Ps for categorical variables were estimated from χ^2^ tests. P for the comparison of median ages was esimated from Wilcoxon Rank-Sum test and Ps for other continuous variables were estimated from t-tests.

2 Histology, lymph node metastasis, grade, MIB1, P53 expression and DNA ploidy index were unknown for 11, 48, 2, 7, 106, 121, 230 and 1 cases, respectively.

3 Data on nuclear are, perimeter and DNA shape were available for only 353 cases.

4 Mean ±standard deviation.

Table 3 shows the associations between select known breast cancer risk factors and breast cancer subtypes defined by ER and PR status. Age at menarche, parity, and menopausal status were not significantly different by ER or PR status, after after adjusting for age at diagnosis. Women with a family history of breast cancer were more likely to have ER−&PR− tumors than women without a family history (OR, 1.43; 95% CI, 0.91–2.26). Given the strong association between family history and early onset of breast cancer, we also evaluated this relationship stratified by age (Table 4) and found that the observed association was limited to women who were diagnosed before age 50 (OR, 2.79; 95% CI, 1.34–5.81). There was no association between family history and hormone receptor status among cases diagnosed after age 50. Results were similar when breast cancer subtypes were defined by ER status alone. However, when breast caner subtypes were defined by PR status alone, there was no significant association in any of the age stratum.

**Table 3:** Association between select breast cancer risk factors and hormone receptor status.

	N of	N of	*ER−&PR− vs. ER+/PR+*	
	ER+/PR+	ER−&PR−	OR (95% CI)^1^	*P* 1
**Age at menarche**				
≤12	130	38	1.00	
13–14	209	62	1.02 (0.65–1.63)	0.92
≥15	155	40	0.90 (0.54–1.52)	0.70
**Parity**				
Nulliparous	94	24	1.00	
1–2 children	219	57	0.98 (0.57–1.68)	0.93
3+ children	162	42	1.03 (0.59–1.81)	0.92
**Menopausal status**				
Premenopausal	108	32	1.00	
Peri-menopausal	57	11	0.73 (0.33–1.61)	0.44
Postmenopausal	339	98	1.32 (0.64–2.74)	0.45
**Family history**				
No	413	107	1.00	
Yes	91	34	1.43 (0.91–2.26)	*0.12*

1 Results were estimated from case-only logistic regressions with adjustment for age at diagnosis.

**Table 4:** Family history and breast cancer hormone receptor status by age at diagnosis.

		ER−&PR− vs. ER+/PR+			ER− vs. ER+			PR− vs. PR+	
	N of			N of			N of		
	ER+/PR+ tumors			ER+ tumors			PR+ tumors		
	/ER−&PR− tumors^1^	OR (95% CI)^2^	*P* 2	/ER− tumors^1^	OR (95% CI)^2^	*P* 2	/PR− tumors^1^	OR (95% CI)^2^	*P* 2
Among <50 years old									
Family history									
o	98/19	1.00		97/22	1.00		74/35	1.00	
Yes	40/21	2.79 (1.34–5.81)	*0.006*	39/23	2.66 (1.32–5.39)	*0.006*	36/24	1.40 (0.72–2.72)	*0.32*
Among 50+ years old									
Family history									
No	315/88	1.00		310/98	1.00		228/153	1.00	
Yes	51/13	0.91 (0.48–1.76)	*0.79*	50/15	0.95 (0.51–1.76)	*0.87*	38/20	0.79 (0.44–1.40)	*0.42*

1 Subjects with missing information of ER, PR, or family history were removed from analyses.

2 Results were estimated from case-only logistic regressions with adjustment for age at diagnosis.

Given our prior finding of DNA ploidy as an indepdent prognostic factor for overall survival of breast cancer patients [11], we further classified ER−&PR− tumors by their DNA index and evaluated the association of family history and these tumor subtypes (Table 5). Family history was more pronouncedly associated with diploid/near-diploid ER−&PR− tumors (OR, 2.17; 95% CI, 0.99–4.74) than aneuploid ER−&PR− tumors (OR, 1.23; 95% CI, 0.73–2.08). Such difference in association was also observed when limiting to cases diagnosed before age 50.

**Table 5:** Family history and breast cancer subtypes further stratified by DNA ploidy.

		*Diploid & near-diploid*			*Aneuploid*		
	N of	*ER−&PR−*			*ER−&PR−*		
	ER+/PR+ tumors^1^	N^1^	OR (95% CI)^2^	*P* 2	N^1^	OR (95% CI)^2^	*P* 2
Family history							
No	413	21	1.00		86	1.00	
Yes	91	11	2.17 (0.99–4.74)	*0.053*	23	1.23 (0.73–2.08)	*0.43*
Among <50 years old							
Family history							
No	98	3	1.00		16	1.00	
Yes	40	7	5.87 (1.43–24.06)	*0.014*	14	2.21 (0.97–5.00)	*0.058*

1 Subjects with missing information of ER, PR, or family history were removed from analyses.

2 Results were estimated from case-only logistic regressions with adjustment for age at diagnosis.

## Discussion

In a population-based study of breast cancer patients from Spain, we observed an increase in the proportion of ER−&PR− breast cancer among women with a family history of the disease, and such increase was limited to cases under 50 years of age.

The present study was conducted in Galicia, a region located in the northwest part of Spain, whose history has been defined by mass emigration especially to Latin America. Galicia has been the Spanish region that contributed most to Latin America's emigration in the 1800s and 1900s [15]. In the United States, Hispanics are a diverse and growing community that represents 12% of the US population [16]. Hispanic ethnicity, as defined by the Office of Management and Budget in 1978, refers to persons or descendants of people from Latin American countries or other Spanish cultures. Under this definition, Hispanics are culturally and genetically a heterogeneous group [17]. In Latin America, each country has its own demographic and genetic structure, with its own distinct migration history between regions. All Hispanics are basically tri-hybrid, i.e., their ancestral populations being European, African, and Native American with the European contribution usually being the highest, although this varies to a degree [18]. The fact that Galicia has been the European state with the highest emigration to Latin America in the 1800s and 1900s makes Galicia a likely contributor of the European ancestry to Hispanics in the United States. In addition, the Galician population provides an interesting contrast group to Hispanics from the San Luis Valley, Colorado in the United States, many of whom self-identify as being of “Spanish origin” [19].

In general, Hispanic patients with breast cancer tend to have ER-negative tumors more frequently than non-Hispanic white women [20], [21]. Using 1990 to 2001 data from 11 population-based cancer registries that participated in the SEER program, Dunnwald et al. [22] reported that 19% of non-Hispanic white breast cancer cases and 26% of Hispanic white cases were ER−&PR− tumors. The 4-Corners Breast Cancer Study examined women with a family history of breast cancer and showed that Hispanic women had a higher incidence of triple-negative breast cancer, whereas NHW women had a higher incidence of postmenopausal hormone receptor–positive breast cancer [3]. In this study, ER−&PR− tumors were observed among 22% of patients, a rate comparable to previous reports of Hispanics in the United States [1], [22]. In addition, our findings that ER−&PR− tumors were more likely to be high grade, highly proliferative but had similar tumor size and less lymph node involvement, supports the hypothesis that the presence or absence of ER and PR represents distinct biological entities rather than different stages in the natural history of the disease.

Family history of breast cancer is an important established risk factor of the disease. Most previous studies [23] have found that a positive family history of breast cancer seems to increase risk similarly for ER+ and ER− tumors and similarly for all ER/PR subtypes. In the Multiethnic Cohort Study [1], a family history of breast cancer was similary associated with breast cancer subtypes defined by ER and PR status, with hazard ratios ranging from 1.63 to 1.91 after adjusting for race/ethnicity and other known risk factors of breast cancer. However, Hines et al. [3] found different associations between family history and risk of breast cancer subtypes when examined in Hispanic women and non-Hispanic white women separately, with ORs of 1.89 and 1.41 for ER+ and ER− breast cancer respectively among NHW women and 1.04 and 2.66 among Hispanics. Consequently, in the case-only analysis, women with a family history were found to have a significantly higher proportion of ER− tumors compared with women without a family history among Hispanics, but not among NHWs. This obsevation among Hispanics was consistent with our finding of an association between family history and breast cancer receptor-defined subytpes among women from Spain. Furthermore, both Hines et al. [3] and our study found that results were similar with or without adjusting for PR status, indicating possible differential involvement of ER and PR in the etiology of the disease, or, alternatively that the driving force in the association with family history is ER status and PR status does not really play a role above and beyond that of ER.

An increased risk associated with a positive family history may be attributed to shared genetic factors and environmental exposures. Both Hines et al. [3] and our study found that the association between family history and receptor-negative tumors was stronger among younger women indicates a more important contribution of genetic factors to family history. Hence, the finding of a higher proportion of receptor-negative tumors among women with a family history than those without suggests that hormone-receptor-positive and receptor-negative tumors have different genetic components to their risks. In line with this notion, most breast cancers that occur in women with germline *BRCA1* mutations are ER− and PR− and younger *BRCA1* carriers were significantly more likely to develop an ER− cancer compared with older carriers [24]. Furthermore, breast cancer susceptibility loci identified from genome-wide association studies were also found to confer risk differentially for ER+ and ER− breast cancers [25], [26].

It is unclear why the association between family history and breast cancer hormone receptor status was observed among Hispanics, but not among NHWs. It is possible that genetic susceptibility to breast cancer may differ among ethnic populations. Major differences in gene expression between Hispanics and NHW have also been described [27]. Baumbach et al. [28] presented genetic microarray analysis of 28 paraffin-embedded, triple-negative breast cancer samples from Hispanic, white, and black women. Ethnic-specific expression patterns were observed in both tumor and normal tissue specimens. Significant differential expression of DNA repair pathway genes was observed in tumor samples from all 3 ethnic groups. In another study [29], the gamma-aminobutyric acid A receptor, whose progenitor cells are hypothesized to proliferate within the breast lobules during pregnancy and then are progressively lost during breastfeeding, was expressed at higher levels in Hispanic women compared with age-matched white controls.

The contribution of *BRCA* mutations may be different among Hispanic breast cancer patients. In a population-based multiethnic series of female breast cancer patients [30], *BRCA1* mutation was prevalent in 3.5% of Hispanics but only 2.2% of non-Hispanic whites, suggesting differential contribution of *BRCA1* mutations to familial breast cancers in Hispanic women. Novel sequence variants in *BRCA1* and *BRCA2* have been found in Spanish families with multiple cases of breast and ovarian cancer [31], [32], [33]. Founder effects have been observed in the Galician population for some genetic diseases, including *BRCA1* in familial breast cancer [34]. It has been shown that the *BRCA1* mutation *A330G*, which results in a Arg to Gly change at codon 71 (*R71G*), could have a Galician origin [32], [34]. This mutation has been observed in families in diverse geographical locations (Spain, Caribbean, France, United Kingdom) which all have a Spanish origin, and it co-segregates with cancer in those families [35]. Families inheriting this mutation were not recently related, and most of them can trace their history to the Spanish colonization period, suggesting that the families studied shared a common ancestry with *BRCA1 A330G* being a founder mutation of Spanish origin. In the largest study of high-risk Hispanic families in the United States [36], *185delAG*, a founder mutation seen in ∼1% of individuals of Ashkenazi Jewish ancestry, was found to be the most common deleterious *BRCA* mutation and share the same haplotype as a reference Ashkenazi Jewish population. Haplotype analyses of additional recurrent *BRCA1* mutations also suggest founder effects, with four of six mutations seen almost exclusively in families with Latin American/Caribbean or Spanish ancestry.

Aneuploidy, the numerical chromosomal aberrations, is one of the most common abnormalities in cancer [37]. It has been suggested that numerical chromosome changes and ploidy shifts are pathogenetically important rather than only epiphenomena in carcinogenesis. The finding that the effect of FH was limited to diploid or near diploid ER−&PR− tumors suggests distinct biological mechanisms linking family history to receptor-negative tumors.

The present study has a number of limitations. First, we were unable to collect detailed information of patients' family, such as the number of affected relatives and age at diagnosis of the affected relatives. Second, we did not collect information on sibship size such that risks of family history could be calculated with adjustment for this potential confounder. We recognize the potential implications of this missing piece of information. Third, our definition of family history considered all first and second-degree relatives and recall of cancer history is generally more accurate for first-degree than second-degree relatives [38]. Therefore, more misclassification may have occurred, biasing our estiamtes towards null. In addition, the lack of a cancer-free control group may limit our ability to generalize these results to the general population. Finally, even though we were able to collect relevant clinic-pathological data of breast cancer providing additional information to the literature, such data were only available for a subset of our participating patients.

In conclusion, our analysis of a Hispanic population from Spain demonstrates an increase in the proportion of ER−&PR− breast cancer among women with a family history of the disease, an increase that was limited to cases under 50 years of age. Our results also indicate that the driving force in breast cancer etiological differences may be ER not PR status. These results complement emerging evidence that relationships for genetic susceptibility loci also vary by expression levels of markers in tumors [39]. Our results support the view that there may be more than one type of breast cancer from an etiological perspective, and specifically support the hypothesis that hormone receptor negative tumors may have different etiologies from hormone receptor positive tumors.
